# Inhibition of esophageal-carcinoma cell proliferation by genistein via suppression of JAK1/2-STAT3 and AKT/MDM2/p53 signaling pathways

**DOI:** 10.18632/aging.103019

**Published:** 2020-04-10

**Authors:** Jing Gao, Rongmu Xia, Jianbo Chen, Jing Gao, Xianyang Luo, Chunlin Ke, Caihong Ren, Jiayi Li, Yanjun Mi

**Affiliations:** 1 Department of Head and Neck Surgery, The First Affiliated Hospital of Xiamen University, Teaching Hospital of Fujian Medical University, Xiamen 361003, Fujian Province, P.R. China; 2 School of Medicine, Xiamen University, Xiamen 361102, Fujian Province, P.R. China; 3 Department of Medical Oncology, Cancer Center, Xiamen Key Laboratory of Antitumor Drug Transformation Research, The First Affiliated Hospital of Xiamen University, Teaching Hospital of Fujian Medical University, Xiamen 361003, Fujian Province, P.R. China; 4 Department of Radiation Oncology, The First Affiliated Hospital of Fujian Medical University, Fuzhou 350005, Fujian Province, P.R. China; 5 Department of Pathology, The First Affiliated Hospital of Fujian Medical University, Fuzhou 350005, Fujian Province, P.R. China

**Keywords:** esophageal carcinoma, genistein, proliferation, JAK1/2-STAT3 pathway, AKT/MDM2/p53 pathway

## Abstract

Esophageal carcinoma (EsC) is a clinically challenging neoplastic disease. Genistein, a natural isoflavone product, has anti-tumor properties. Through *in vitro* and *in vivo* studies, we found that genistein suppressed EsC cell proliferation in a time- and concentration-dependent manner. In addition, genistein markedly promoted apoptosis and arrested cell cycle at the G0/G1 phase in a concentration-dependent manner. Furthermore, high concentrations of genistein have no adverse effect on normal esophageal epithelial cells. Mechanistically, genistein treatment strikingly reduced the expression of cell cycle-associated genes, and up-regulated the expression of cell apoptosis-related genes in EsC cells. Additionally, genistein dramatically decreased epidermal growth factor receptor (EGFR) expression and attenuated its down-stream signaling molecules STAT3, MDM2, Akt and JAK1/2 phosphorylation, resulting in inhibited nuclear translocation of STAT3 and MDM2, thereby inhibiting the JAK1/2-STAT3 and AKT/MDM2/p53 signaling pathways. In xenograft nude mice, genistein administration strikingly impaired tumor growth in a dose-dependent manner. Moreover, similar disturbances in molecular mechanisms were observed *in vivo*. Taken together, genistein suppressed the JAK1/2-STAT3 and AKT/MDM2/p53 signaling pathways by decreasing EGFR expression, leading to cell apoptosis, cell cycle arrest, and proliferation inhibition in EsC cells. Our findings suggest that genistein may be a promising alternative adjuvant therapy for patients with EsC.

## INTRODUCTION

Esophageal carcinoma (EsC) is a malignant tumor originating from the mucosal epithelium of the esophagus. In 2015, there were approximately 477,900 new cases of esophageal cancer and 37,500 related deaths in China, accounting for 11.13% and 13.33% of the tumor incidence and mortality, respectively [1]. The prevention and treatment of EsC in China is a great challenge.

Because of the relatively insidious onset of EsC and the lack of early specific symptoms, most patients are diagnosed at a mid-term or advanced stage. The 5-year survival rate of patients with EsC after treatment is only 10%–25%, and the 5-year survival rate of EsC patients at an advanced stage is less than 10% [2, 3]. Chemotherapy is the standard therapy for patients with advanced EsC who cannot receive surgical treatment, and the therapeutic effect depends on the sensitivity of the tumor cells to the chemotherapy drugs. However, chemo-resistant tumors are common, resulting in the failure of chemotherapy [4, 5].

Flavonoids, including genistein, daidzein and soy flavonoids are extracted from plant phenols [6, 7]. Genistein is the simplest isoflavone compound biosynthesized by legumes and is widely distributed in plants, such as soybeans, Scutellaria, and Pueraria. Genistein has a similar structure as estrogen. Through binding to the estrogen receptor, genistein exerts strong activity, and it is considered to be an active phytoestrogen [8, 9]. An increasing number of studies have shown that genistein is involved in inducing cancer cell apoptosis, inhibiting cell proliferation, suppressing angiogenesis and inhibiting colorectal cancer metastasis [10]. Clinical trials have confirmed that genistein markedly reduced the positive rate of Ki-67 in postmenopausal women with breast cancer [11]. Therefore, genistein may be a promising agent for cancer prevention. However, little is known about the mechanism of genistein in the treatment or prevention of esophageal cancer.

The occurrence and development of esophageal cancer is significantly associated with the up-regulation of tyrosine kinase expression or activity [12]. Genistein is a protein tyrosine kinase inhibitor [13, 14]. Tyrosine phosphorylation activity was strikingly attenuated following treatment using genistein alone or in combination with the tyrosine kinase inhibitor AG556, leading to inhibition of ultra-rapidly activating delayed rectifier K(+) current of human atria [15]. In addition, genistein increases the ratio of Bax to Bcl-2 by inducing P53 expression, promoting sensitivity to radiotherapy in esophageal cancer [16]. A large-scale retrospective analysis of populations in northwestern China’s Xinjiang Province showed a significant negative correlation between the consumption of soy products (abundant in genistein) and the risk of esophageal cancer [17]. We hypothesized that genistein may inhibit the development of esophageal cancer by inhibiting the activity of tyrosine kinases. Fully understanding the mechanisms of genistein in esophageal cancer may be helpful in finding potential therapeutic drugs.

## RESULTS

### Genistein suppresses the proliferation of esophageal cancer cells

**Figure 1:**
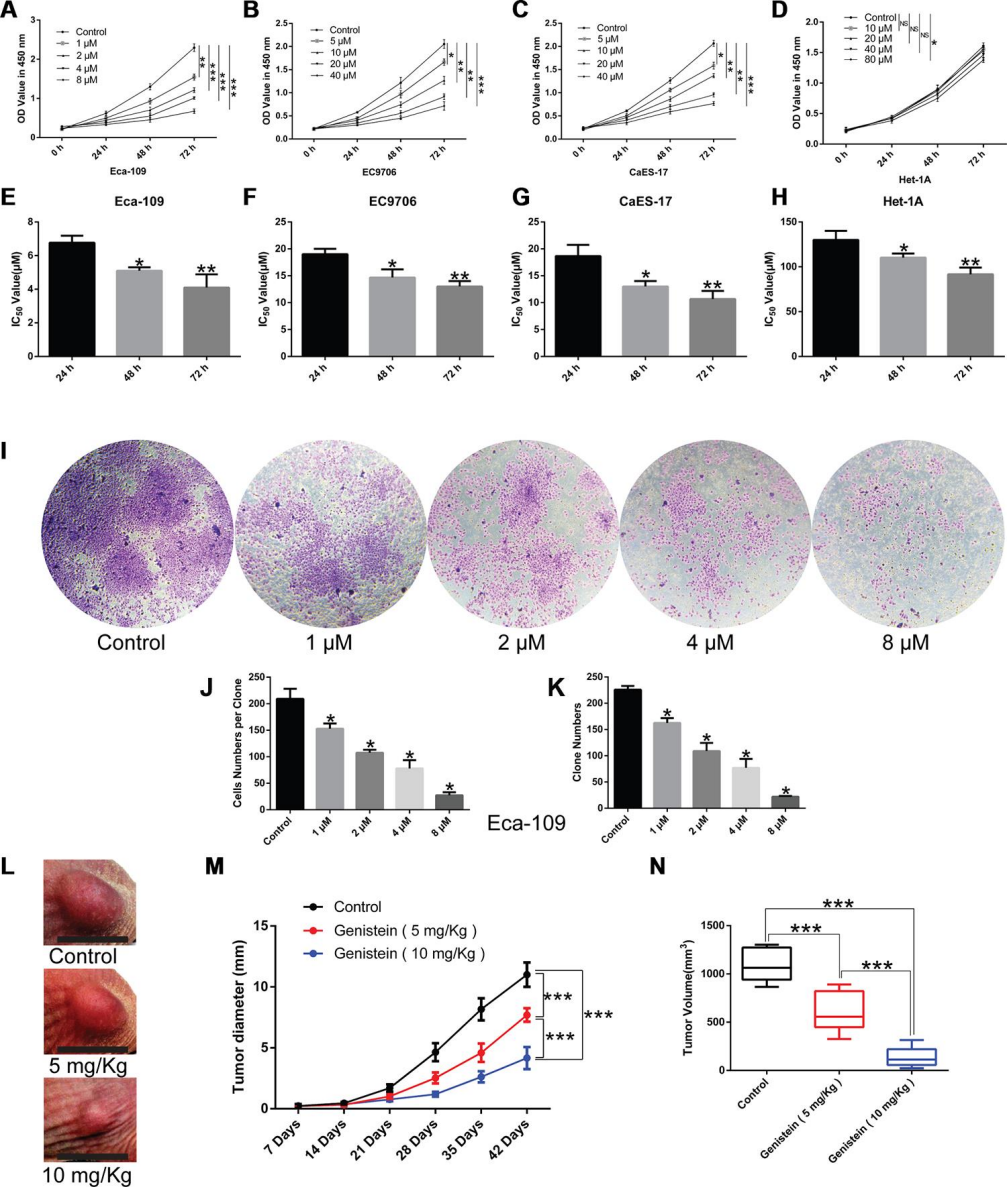
**Genistein inhibits the proliferation of esophageal cancer cells.** A CCK-8 assay was performed to measure the effect of genistein on the proliferation of the (**A**) Eca-109, (**B**) EC9706, and (**C**) CaES-17 esophageal cancer cell lines, and (**D**) the human esophageal epithelial cell line Het-1A. The IC_50_ of genistein in (**E**) Eca-109, (**F**) EC9706, (**G**) CaES-17 and (**H**) Het-1A cells at 24 h, 48 h and 72 h, respectively. (**I**) A clone formation assay was performed to detect the proliferative ability of Eca-109 cells treated with various concentrations of genistein for 9 d. Magnification, 40×. (**J**) Cell numbers per clone. (**K**) Quantification of clone numbers in each well (6-well plate). *in vivo*, the effect of different concentrations of genistein (5 mg/kg, 10 mg/kg) on (**L**) the cell growth of Eca-109 cells, (**M**) tumor diameter and (**N**) tumor volume (n = 6 per group). All *in vitro* experiments were independently repeated in triplicate. Results were analyzed using one-way ANOVA with Dunnett’s test (*in vitro*) or Fisher’s least significant difference (LSD) test (*in vivo*). Data are presented as the mean ± SD. *P<0.05; **P<0.01; ***P<0.001; NS, not significant.

To determine the effect of genistein on the proliferation of esophageal cancer cell lines, a CCK-8 assay was conducted. As shown in Figure 1, genistein significantly reduced EsC cell proliferation in a concentration- and time-dependent manner (Figure 1A–1C, P<0.05), but a high concentration of genistein (40 μM; 72 h) had no adverse effect on the cell viability of human esophageal epithelial Het-1A cells (Figure 1D) compared with matched un-treated cells, indicating that the cytotoxicity of genistein is selective for esophageal cancer cells. The half-maximal inhibitory concentration (IC50) values for the Eca-109, EC9706, and CaES-17 esophageal cancer cell lines as well as Het-1A cells treated with genistein for 48 h were 5 μM, 15 μM, 12 μM and 125 μM, respectively (Figure 1E–1H, P<0.05). To further test the inhibitory effect of genistein on EsC cells, we evaluated clone formation after the treatment of Eca-109 cells with various concentrations of genistein (1 μM, 2 μM, 4 μM and 8 μM), and genistein inhibited clone formation in a concentration-dependent manner (Figure 1I–1K, P<0.05). Moreover, consistent with the *in vitro* results, genistein administration remarkably suppressed tumor growth in EsC cell xenografts *in vivo* (Figure 1L–1N, P<0.001).

### Genistein induces apoptosis in esophageal cancer cells and arrests cell cycle in G0/G1 phase

**Figure 2:**
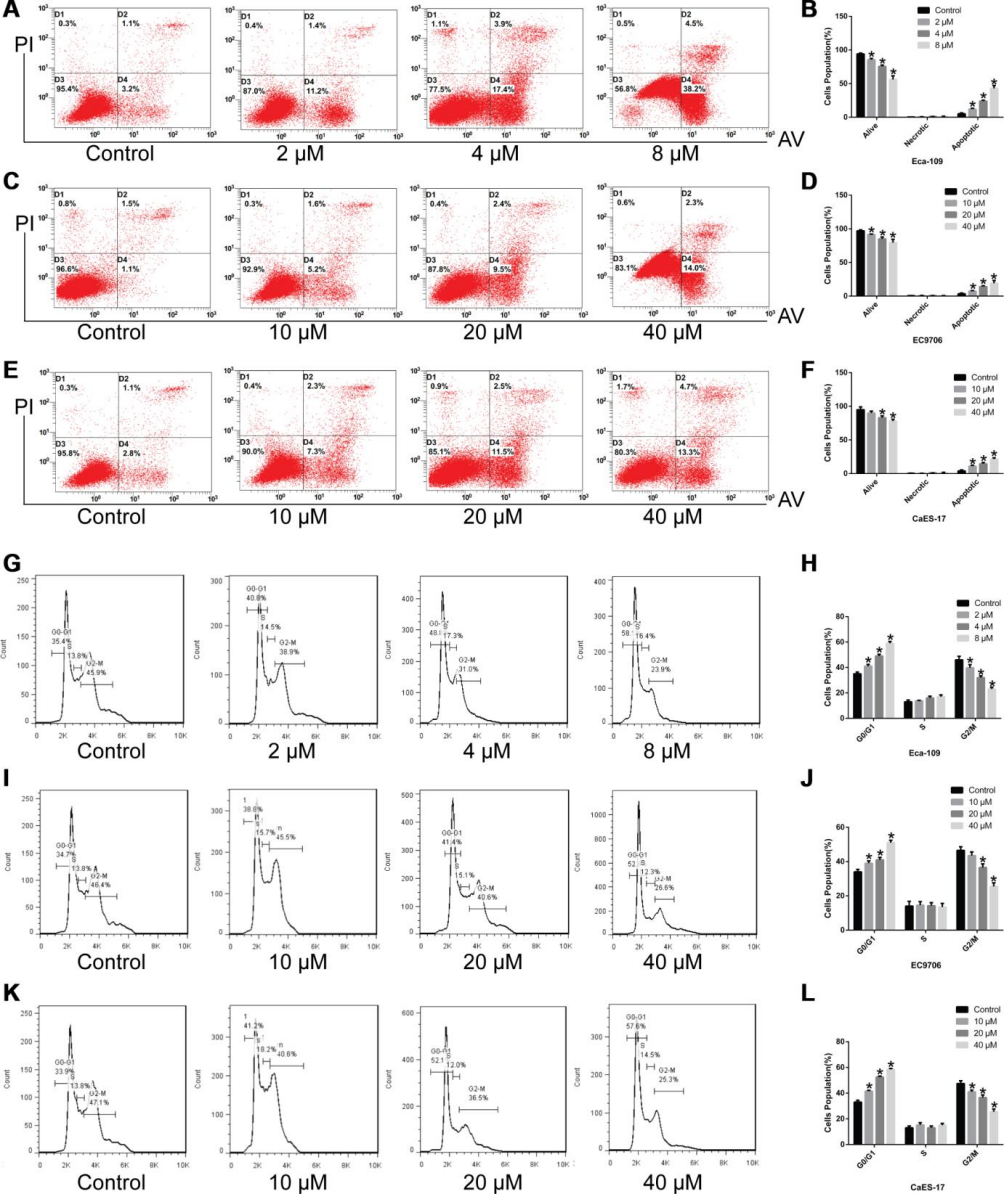
**Genistein induces apoptosis in EsC cells and arrests cell cycle in G0/G1 phase.** The cell apoptosis of (**A**, **B**) Eca-109, (**C**, **D**) EC9706 and (**E**, **F**) CaES-17 cells treated with various concentrations of genistein for 24 h was detected using flow cytometry. The cell cycle of (**G**, **H**) Eca-109, (**I**, **J**) EC9706 and (**K**, **L**) CaES-17 cells treated with different concentrations of genistein for 24 h was detected using flow cytometry. All experiments were independently repeated in triplicate. Data are analyzed using one-way ANOVA with Dunnett’s test and presented as the mean ± SD. *P<0.05.

To elucidate the mechanism of genistein-induced cytotoxicity in esophageal cancer cells, cell apoptosis and cell cycle assays were carried out on Eca-109, EC9706, CaES-17 and human esophageal epithelial Het-1A cells. We found that genistein induced apoptosis (Figure 2A–2F, P<0.05) and caused G0/G1 cell cycle arrest (Figure 2G–2L, P<0.05) in EsC cells in a concentration-dependent manner, but when the concentration of genistein was below 50 μM (genistein treatment for 24 h), it had no adverse effects in Het-1A cells (Supplementary Figure 1A–1D).

### Genistein inhibits the expression of cell cycle-related genes and promotes the expression of cell apoptosis-associated genes

**Figure 3:**
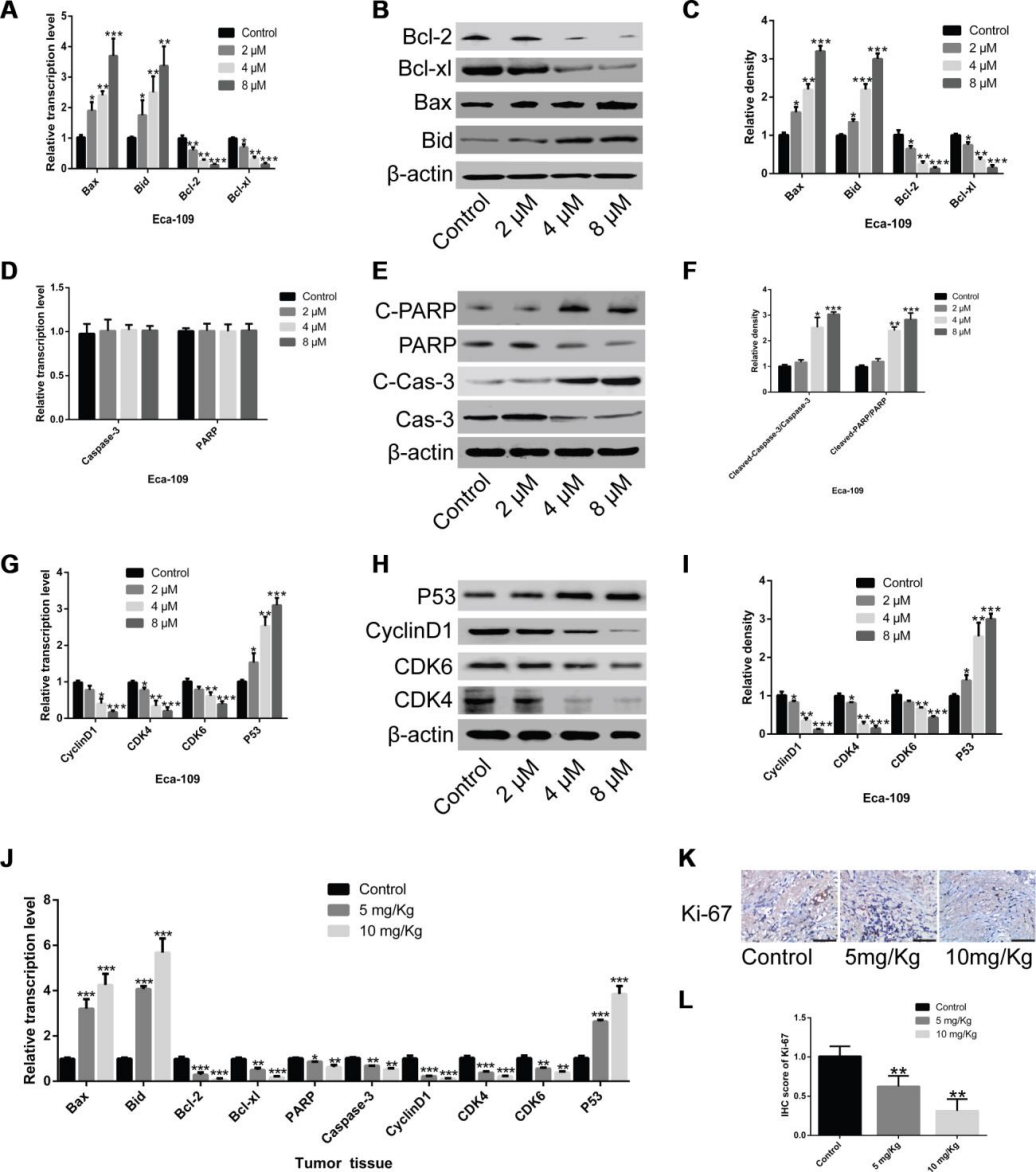
**Genistein inhibits the expression of cell cycle-related genes and promotes the expression of cell apoptosis-associated genes.** (**A–C**) The mRNA and protein levels of Bcl-2, Bcl-xl, Bax and Bid in Eca-109 cells treated with genistein for 72 h were determined through qPCR and western blotting. (**D**) The mRNA levels of PARP and Caspase-3 in Eca-109 cells treated with genistein for 72 h. (**E**, **F**) The protein levels of PARP, Caspase-3, cleaved PARP and cleaved Caspase-3 in Eca-109 cells treated with genistein for 72 h. (**G–**I) The mRNA and protein levels of P53, CyclinD1, CDK6 and CDK4 in Eca-109 cells treated with genistein for 72 h. (**J**) The mRNA and protein levels of Bax, Bid, Bcl-2, Bcl-xl, PARP, Caspase-3, CyclinD1, CDK4, CDK6 and P53 in xenograft tumor tissues were validated using qPCR and western blotting. Tumor-bearing nude mice were treated using different concentrations of genistein (5 mg/kg or 10 mg/kg) through gavage every 2 d (total time, 42 d; n = 6 per group). (**K**, **L**) Ki-67 expression in xenograft tumor tissues was tested through immunohistochemistry. Scale bar, 100 μm. All experiments were independently repeated in triplicate. Data are analyzed using one-way ANOVA with Dunnett’s test and presented as the mean ± SD. *P<0.05; **P<0.01; ***P<0.001; C-, cleaved.

To understand the molecular mechanisms of genistein in EsC cells, we examined the expression of cell apoptosis-associated genes and cell cycle-related genes. Genistein treatment induced an up-regulation of Bax and Bid in Eca-109 cells, and caused the down-regulation of bcl-2 and bcl-xl at the transcriptional and translational levels (Figure 3A–3C, *P*<0.05). The cleavage of PARP and caspase-3 activity were strikingly elevated (Figure 3D–3F, *P*<0.05). Additionally, the expression of CyclinD1, CDK4 and CDK6 were significantly decreased in Eca-109 cells treated with genistein, whereas P53 expression was markedly increased (Figure 3G–3I, *P*<0.05). However, 50 μM of genistein treatment for 72 h did not affect the expression of the above-mentioned genes in human esophageal epithelial Het-1A cells compared with control cells (Supplementary Figure 1G–1I). In xenograft tumor tissues, similar gene expression changes in EsC cells were observed in nude mice, and the expression of PARP and Caspase-3 were substantially down-regulated (Figure 3J, *P*<0.05). Immunohistochemistry revealed that genistein administration significantly reduced the positive rate of Ki-67 in xenograft tumor tissues in a dose-dependent manner compared with the untreated group (Figure 3K and 3L, *P*<0.05).

### Genistein reduces mitochondrial membrane potential and increases reactive oxygen species (ROS) levels in Eca-109 cells

**Figure 4:**
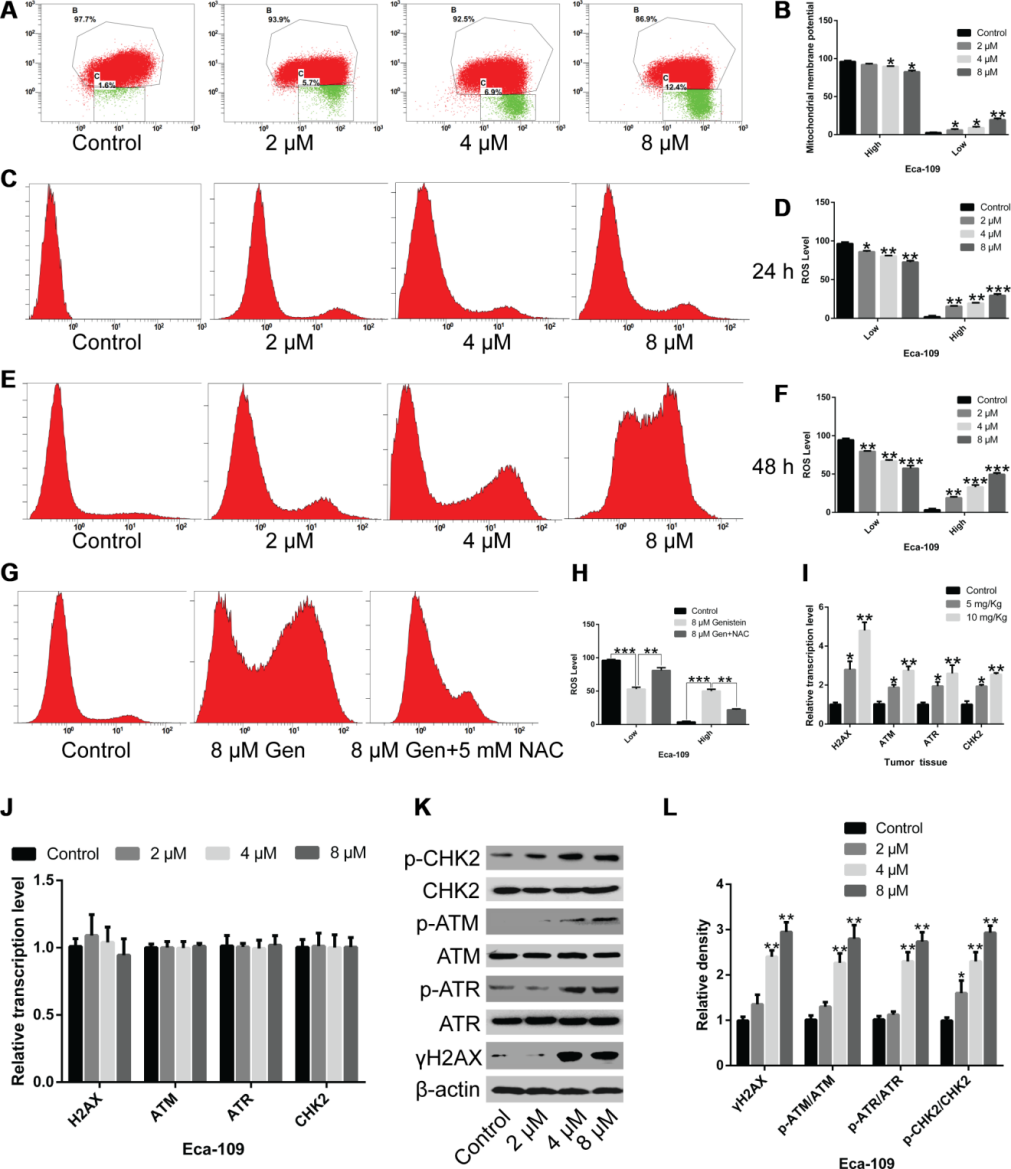
**The effects of genistein on mitochondrial membrane potential, ROS levels and the activation of DNA damage response kinases in Eca-109 cells.** (**A**, **B**) Mitochondrial membrane potential in Eca-109 cells treated with genistein for 24 h was detected through flow cytometry. ROS levels in Eca-109 cells treated with genistein for (**C**, **D**) 24 h or (**E**, **F**) 48 h were detected through flow cytometry. (**G**, **H**) The ROS levels in Eca-109 cells treated with 5 mM NAC for 6 h, followed by genistein treatment for 48 h. (**I**) The mRNA levels of γH2AX, ATM, ATR and CHK2 in xenograft tumor tissues were measured through qPCR (n = 6 per group). (**J**) The mRNA levels of γH2AX, ATM, ATR and CHK2 were analyzed through qPCR. (**K**, **L**) The protein expression of γH2AX, ATM, ATR, CHK2, p-ATM, p-ATR and p-CHK2 in Eca-109 cells treated with genistein for 72 h was measured through western blotting. All experiments were independently repeated three times. Data are analyzed using one-way ANOVA with Dunnett’s test and are presented as the mean ± SD. *P<0.05; **P<0.01; ***P<0.001; NAC, N-acetylcysteine; Gen, genistein.

To understand the mechanism through which genistein affects the cyclical distribution of esophageal cancer cells and induces apoptosis, mitochondrial membrane potential was detected in Eca-109 cells treated with genistein. The results show that the mitochondrial membrane potential of Eca-109 cells was significantly decreased after genistein treatment (Figure 4A and 4B, *P*<0.05) compared with un-treated cells. Moreover, genistein facilitated ROS production in esophageal cancer cells in a time and dose-dependent manner (Figure 4C–4F, *P*<0.05). When Eca-109 cells were pretreated with 5 mM of N-acetylcysteine (NAC), a ROS inhibitor, for 6 h following treatment with genistein (8 μM), ROS levels were reduced compared with Eca-109 cells treated with genistein alone (Figure 4G and H, *P*<0.01), suggesting that genistein promoted ROS production. In addition, western blot analysis confirmed that genistein treatment activated the phosphorylation of histone protein H2AX, ataxia-telangiectasia mutated protein kinase (ATM), Rad3-related protein kinase (ATR) and checkpoint kinase 2 (CHK2) in Eca-109 cells, while no significant difference was observed in the mRNA levels of H2AX, ATM, ATR, and CHK2 (Figure 4J–4L) compared with untreated Eca-109 cells. However, compared with control cells, the expression levels of H2AX, ATM, ATR, and CHK2 were not different in human esophageal epithelial Het-1A cells subjected to a 50 μM genistein treatment at the translational and/or transcriptional level (Supplementary Figure 1G–1I). *in vivo*, the mRNA levels of H2AX, ATM, ATR and CHK2 were remarkably up-regulated in the tumor tissues of mice following intragastric administration of genistein, compared with the tumor tissues of untreated mice (Figure 4I, *P*<0.05), indicating that long-term treatment has a significant effect on EsC cells *in vivo*. Collectively, genistein may affect mitochondrial membrane potential and intracellular ROS production in EsC cells, and regulate the expression of cell cycle-related genes and apoptosis-associated genes, leading to cell apoptosis and cell cycle arrest.

### Genistein represses the JAK/STAT3 and AKT/MDM2/p53 signaling axis by decreasing STAT3 and MDM2 phosphorylation and restricting their nuclear translocation

**Figure 5:**
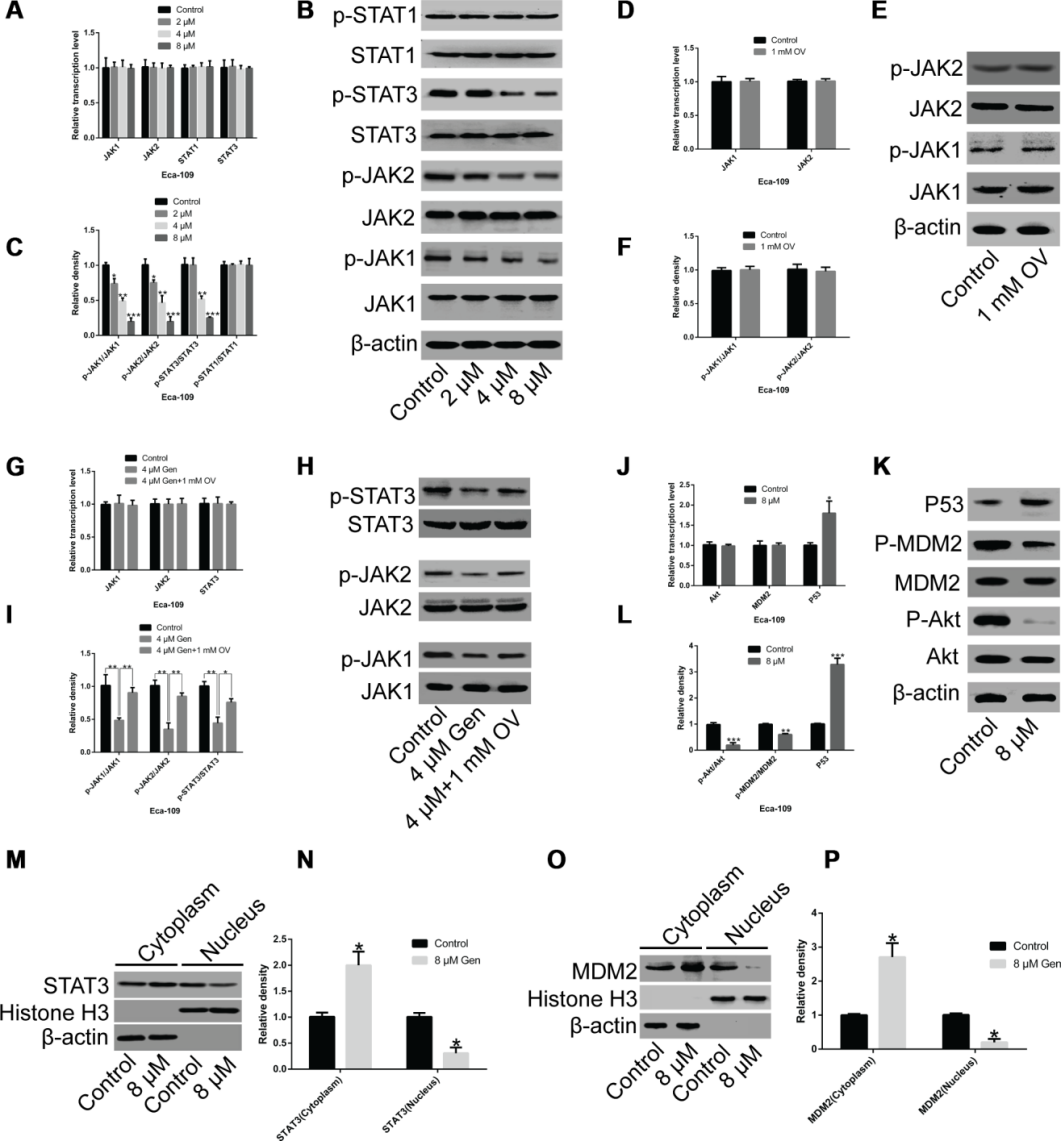
**Genistein inhibits protein phosphorylation and nuclear translocation.** (**A**) The mRNA level of JAK1, JAK2, STAT1 and STAT3 were analyzed through qPCR in Eca-109 cells treated with different concentrations of genistein (2 μM, 4 μM, 8 μM) for 72 h. (**B**, **C**) The protein levels of JAK1, JAK2, STAT1, STAT3, p-JAK1, p-JAK2, p-STAT1 and p-STAT3 were measured through western blotting. (**D**) The mRNA levels of JAK1 and JAK2 in Eca-109 cells treated with 1 mM OV for 72 h. (**E**, **F**) The protein levels of JAK1, JAK2, p-JAK1 and p-JAK2 in Eca-109 cells treated with 1 mM OV for 72 h. (**G**) The mRNA levels of JAK1, JAK2 and STAT3 in Eca-109 cells treated with 1 mM OV or in combination with 4 μM genistein for 72 h. (**H**, **I**) The protein levels of JAK1, JAK2, STAT3, p-JAK1, p-JAK2 and p-STAT3 in Eca-109 cells treated with 1 mM OV or in combination with 4 μM genistein for 72 h. (**J**) The mRNA levels of Akt, MDM2 and Akt in Eca-109 cells treated with 8 μM genistein for 72 h. (**K**, **L**) The protein levels of P53, MDM2, p-MDM2, Akt and p-Akt in Eca-109 cells treated with 8 μM genistein for 72 h. (**M**, **N**) STAT3 and (**O**, **P**) MDM2 levels in the nucleus and cytoplasm of Eca-109 cells treated with 8 μM genistein for 72 h. All experiments were independently repeated three times. Data are analyzed using one-way ANOVA with Dunnett’s test and presented as the mean ± SD. *P<0.05; **P<0.01; ***P<0.001; Gen, genistein; OV, sodium orthovanadate; p-, phosphorylated.

To uncover the mechanism through which genistein affects cyclical distribution and apoptosis in esophageal cancer cells, we tested the expression levels and phosphorylation levels of JAK1, JAK2, STAT1 and STAT3 in Eca-109 cells. In Figure 5, compared with un-treated cells, the phosphorylation levels of JAK1, JAK2 and STAT3 were significantly decreased after genistein treatment, but not those of STAT1 (Figure 5A–5C). Furthermore, 1 mM sodium orthovanadate (OV) treatment for 72 h (a protein tyrosine phosphatase inhibitor) did not affect the tyrosine phosphorylation of JAK1 and JAK2 (Figure 5D–5F, *P*<0.05), suggesting that tyrosine phosphorylation of JAK1 and JAK2 in EsC cells is physiologically saturated. The downregulation of JAK1, JAK2 and STAT3 phosphorylation induced by genistein treatment could be partially rescued by OV (Figure 5G–5I, *P*<0.05). Additionally, compared with un-treated Eca-109 cells, genistein treatment did not affect the expression of Akt and MDM2, but greatly down-regulated Akt and MDM2 phosphorylation; P53 expression was significantly up-regulated in Eca-109 cells treated with genistein (Figure 5J–5L, *P*<0.05). On analysis of nuclear and cytoplasmic proteins through western blotting, it was found that the levels of STAT3 (Figure 5M and N, *P*<0.05) and MDM2 (Figure 5O and 5P, *P*<0.05) in the nucleus were strikingly reduced after genistein treatment. These data suggest that genistein suppressed the JAK/STAT3 and AKT/MDM2/p53 signaling axis by decreasing STAT3 (not STAT1) and MDM2 phosphorylation and restricting their nuclear translocation.

### A synergistic inhibitory effect of genistein with JAK1 pathway inhibitor GLPG0634 and/or Akt pathway inhibitor MK-2206 on the proliferation of Eca-109 cells *in vitro* and *in vivo*

**Figure 6:**
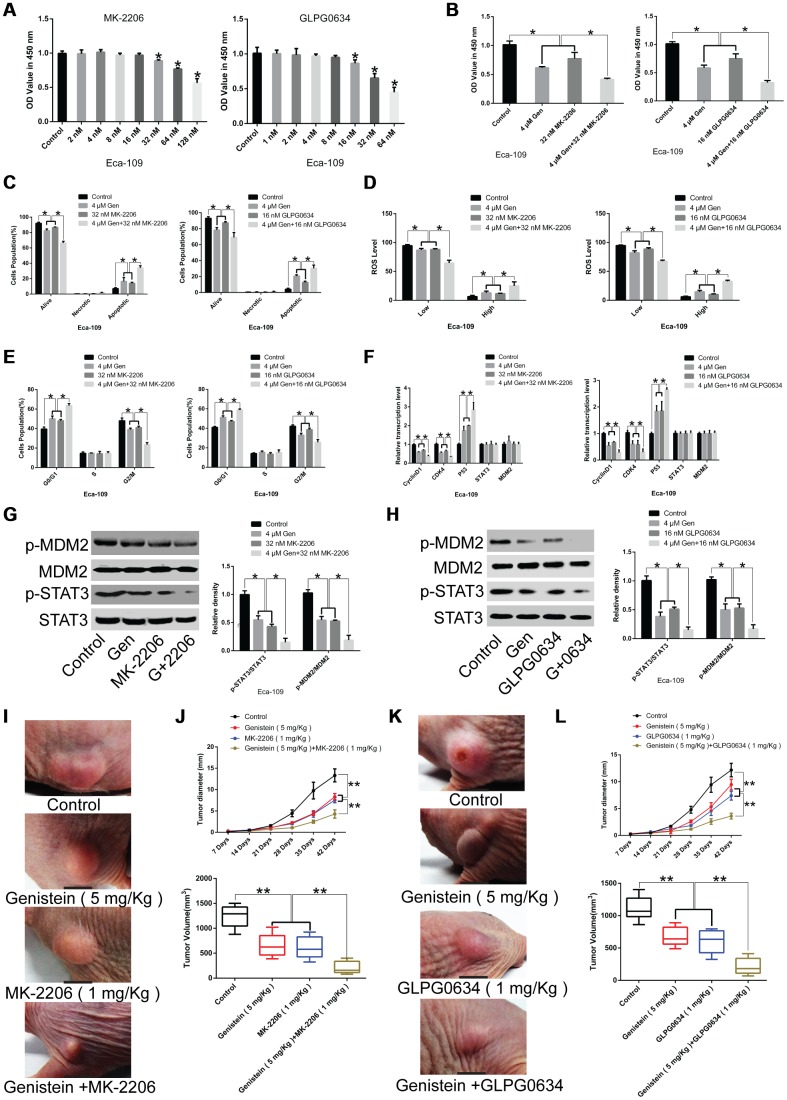
**Synergistic effects of genistein in combination with GLPG0634 or MK-2206 on the proliferation of Eca-109 cells.** (**A**) A CCK-8 assay was conducted to assess the effects of the JAK1 pathway inhibitor GLPG0634 or the Akt pathway inhibitor MK-2206 on the proliferation of Eca-109 cells. (**B**) The effects of genistein (4 μM) in combination with the JAK1 pathway inhibitor GLPG0634 (16 nM) or the Akt pathway inhibitor MK-2206 (32 nM) on the proliferation of Eca-109 cells was measured through a CCK-8 assay. The effects of genistein (4 μM) in combination with GLPG0634 (16 nM) or MK-2206 (32 nM) on (**C**) apoptosis, (**D**) ROS levels and (**E**) the cell cycle in Eca-109 cells were measured through flow cytometry. (**F**) qPCR analysis was performed to quantify the expression of CyclinD1, CDK4, P53, STAT3 and MDM2 in Eca-109 cells co-treated with genistein (4 μM) and GLPG0634 (16 nM) or MK-2206 (32 nM) for 72 h. (**G**) The effects of genistein (4 μM) or MK-2206 (32 nM) or co-treatment on the protein levels of STAT3, p-STAT3, MDM2 and p-MDM2 in Eca-109 cells. (**H**) The effects of genistein (4 μM) or GLPG0634 (16 nM) or co-treatment on the protein levels of STAT3, p-STAT3, MDM2 and p-MDM2 in Eca-109 cells. (**I**, **J**) Genistein (5 mg/kg) treatment alone or in combination with MK-2206 (1 mg/kg) suppresses tumor growth and tumor volume in xenograft nude mice (n = 6 in each group). (**J**) Representative images of tumor volume. (**K**, **L**) Genistein (5 mg/kg) treatment alone or in combination with GLPG0634 (1 mg/kg) inhibits tumor growth and tumor volume in xenograft nude mice (n = 6 per group). (**J**, **L**) Representative images of tumor volume. All *in vitro* experiments were independently repeated three times. Data are analyzed using one-way ANOVA with Dunnett’s test and presented as the mean ± SD. *P<0.05; **P<0.01. Gen or G, genistein. OV, sodium orthovanadate. p-, phosphorylated.

To identify the relationship between genistein and the JAK/STAT3 and AKT/MDM2/p53 pathways, we treated Eca-109 cells with genistein alone or in combination with the JAK1 pathway inhibitor GLPG0634 and/or the Akt pathway inhibitor MK-2206. The JAK1 pathway inhibitor GLPG0634 (16 nM) and the Akt pathway inhibitor MK-2206 (32 nM) were selected for subsequent experiments following CCK-8 assays (Figure 6A). The results show that when Eca-109 cells were co-treated using genistein (4 μM) and the JAK1 pathway inhibitor GLPG0634 (16 nM) or the Akt pathway inhibitor MK-2206 (32 nM), cell proliferation was significantly down-regulated (Figure 6B), while cell apoptosis (Figure 6C) and ROS levels (Figure 6D) were markedly increased, and cell cycle was arrested in the G0/G1 phase (Figure 6E), compared with Eca-109 cells treated with genistein alone. Furthermore, genistein plus the JAK1 pathway inhibitor GLPG0634 and the Akt pathway inhibitor MK-2206 produced substantial decreases in cell viability and facilitated ROS production compared with Eca-109 cells co-treated with genistein and GLPG0634 or genistein and MK-2206, respectively (Supplementary Figure 2).

Next, we investigated the effects of genistein treatment alone or in combination with the JAK1 pathway inhibitor GLPG0634 or the Akt pathway inhibitor MK-2206 on the expression levels of the cell cycle-related gene CyclinD1 and CDK4, P53, and STAT3 as well as MDM2. As expected, CyclinD1 and CDK4 levels were remarkably down-regulated, P53 expression was significantly up-regulated, but no difference was observed in STAT3 and MDM2 levels in genistein and inhibitor co-treated EsC cells, compared with cells treated with genistein alone (Figure 6F). However, genistein in combination with the JAK1 pathway inhibitor GLPG0634 or the Akt pathway inhibitor MK-2206 had an enhanced inhibitory effect on STAT3 and MDM2 phosphorylation in Eca-109 cells in comparison with EsC cells subjected to genistein treatment alone (Figure 6G and 6H, P<0.05).

Notably, consistent results were obtained in a xenograft model, whereby genistein administration (5 mg/kg) strikingly impaired tumor growth. Co-treatment with genistein and the JAK1 pathway inhibitor GLPG0634 (1 mg/kg) or the Akt pathway inhibitor MK-2206 (1 mg/kg) had an enhanced inhibitory effect on tumor growth in comparison with matched control groups (Figure 6I–6L, P<0.01). These data indicate that genistein impaired tumor growth via modulating Akt and JAK-regulated pathways, providing potential adjuvant therapeutic agents for the treatment of EsC.

### Genistein reduces the expression of epidermal growth factor receptor (EGFR) and EGF as well as the reactivity of esophageal cancer cells to EGF

**Figure 7:**
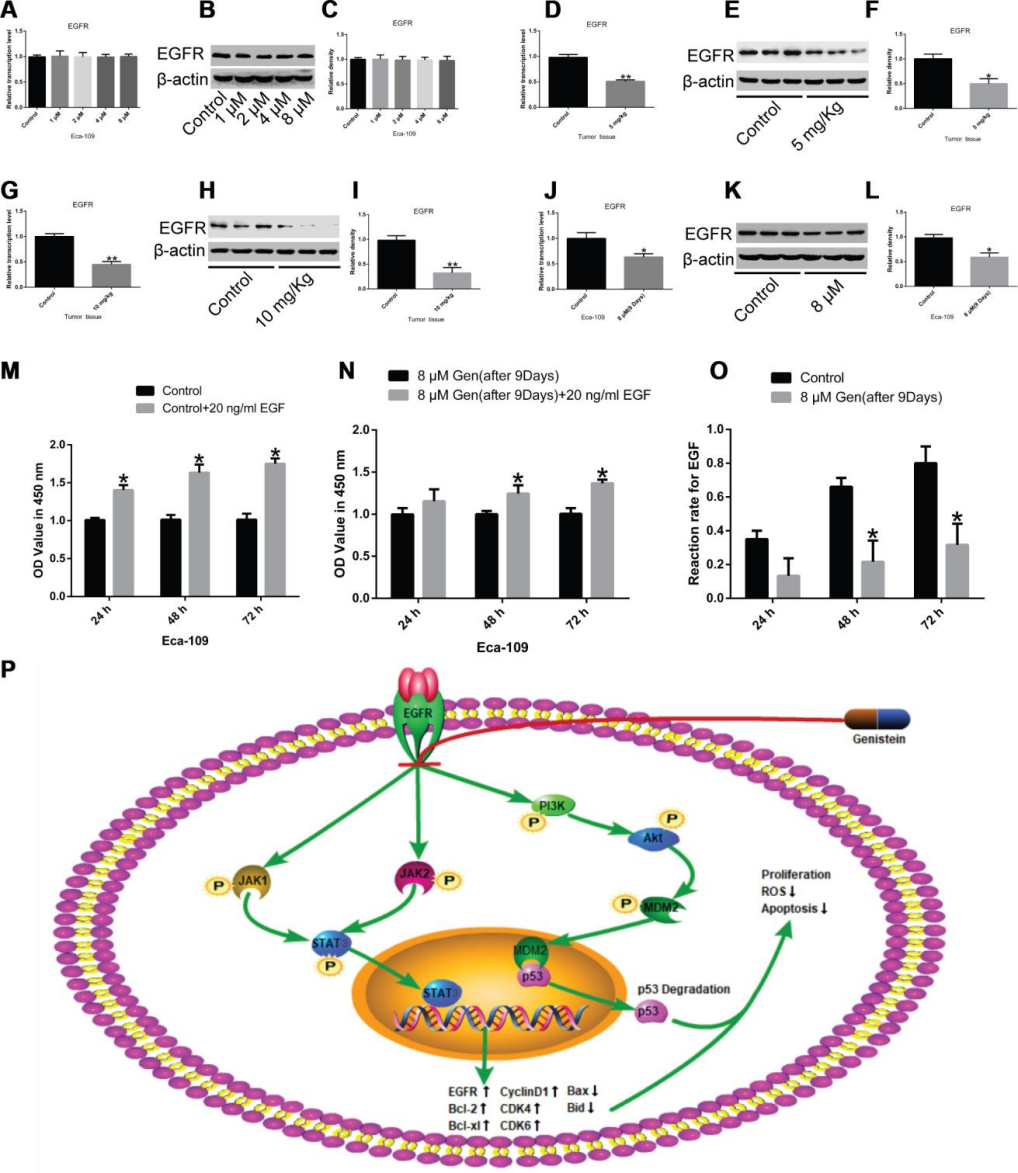
**The effects of genistein on EGFR expression and the reactivity of EsC cells to EGF.** (**A**–**C**) EGFR expression in Eca-109 cells treated with different concentrations of genistein for 72 h was detected through qPCR and western blotting. EGFR expression in xenograft mice treated with (**D**–**F**) 5 mg/kg or (**G**–**I**) 10 mg/kg genistein for 42 d was significantly lower than that in an un-treated group. (**J**–**L**) EGFR expression in Eca-109 cells treated with 8 μM of genistein for 9 d. (**M**) The effect of recombined EGF (20 ng/mL) treatment alone on the proliferation of Eca-109 cells was detected through a CCK-8 assay. (**N**) The cell viability of Eca-109 cells incubated with genistein (8 μM) for 9 d following treatment with recombined EGF (20 ng/mL) for 24 h, 48 h or 72 h. (**O**) The reactivity of Eca-109 cells treated with genistein (8 μM) to recombined EGF. (**P**) A schematic working model to illustrate that genistein inhibits EGFR and its downstream pathways, the PI3K-Akt and JAK1/2-STAT3 signaling pathways, leading to apoptosis and cell cycle arrest in EsC cells. All *in vitro* experiments were independently repeated three times. The difference between two groups was tested using the Student’s t-test, and comparisons among multiple groups were performed using one-way ANOVA with Dunnett’s test. Data are presented as the mean ± SD. *P<0.05; **P<0.01; Gen, genistein.

The receptor tyrosine kinase EGFR plays a vital role in the JAK1/2-STAT3 pathway and the AKT/MDM2/p53 pathway [18, 19]. Therefore, we measured EGFR expression after genistein treatment for 72 h. No significant difference was observed in EGFR expression (Figure 7A–7C). However, in xenograft tumor tissues, we found that genistein treatment strikingly reduced EGFR levels (Figure 7D–7I, P<0.05), suggesting that long-term genistein treatment may inhibit EGFR expression. After genistein treatment for 9 d, we observed that EGFR expression was greatly down-regulated compared with un-treated cells (Figure 7J–7L, P<0.05). EGF treatment alone (20 ng/mL) significantly promoted the proliferation of Eca-109 cells from 24 h compared with un-treated cells (Figure 7M, P<0.05), while genistein (8 μM) treatment for 9 d following treatment with recombinant EGF (20 ng/mL) promoted the proliferation of Eca-109 cells at 48 h compared with cells treated with genistein (8 μM) alone (Figure 7N, P<0.05). The reactivity of esophageal cancer cells to recombined EGF was strikingly attenuated after genistein treatment (8 μM) for 9 d, compared with Eca-109 cells treated with recombined EGF alone (Figure 7O, P<0.05). In conclusion, the inhibition of EGFR suppressed downstream pathways (the JAK1/2/-STAT3 and AKT/MDM2 signaling axis), causing the up-regulation of P53 expression and the down-regulation of cell cycle-related gene expression, thereby arresting cell cycle at the G0/G1 phase and promoting cell apoptosis in EsC cells.

## DISCUSSION

EsC is a clinically challenging neoplastic disease globally. Surgery combined with chemoradiotherapy or neoadjuvant therapy is the typical curative treatment for EsC patients [20]. Because of the extremely aggressive nature of EsC, the overall 5-year survival rate of EsC patients is less than 20% [21, 22]. There is emerging evidence that genistein has anti-tumor effects *in vitro* and *in vivo* [23, 24]. Genistein, known as a natural tyrosine kinase inhibitor [25], inhibits the proliferation, migration and invasion of a variety of tumor cells [26–28]. Intake of genistein-rich soy products can effectively reduce the risk of EsC [16], indicating that genistein may be a novel therapeutic drug for esophageal cancer.

Our results indicated that genistein inhibited the proliferation of various esophageal cancer cells *in vitro*. *in vivo*, genistein administration reduced EsC growth in a dose-dependent manner. Cell growth is commonly impaired by apoptosis or cell cycle arrest [29, 30]. In the current study, flow cytometry revealed that genistein induced apoptosis and G0/G1 arrest in EsC cells in dose-dependent manner. Moreover, the expression of cell cycle-related genes, such as cyclin CDK4/6 and CyclinD1, are necessary to progress the cell cycle from G0/G1 to S phase. The down-regulation of these genes suppressed the progression of cell cycle [31–33]. Our results showed that genistein down-regulated the expression of CDK4/6 and CyclinD1, resulting in EsC cell cycle arrest in the G0/G1 phase. Additionally, the ratio of Bax to Bcl-2 in cells has an effect on cell viability [34, 35], whereby when the ratio of Bax to Bcl-2 increases, cells tend to undergo apoptosis [36, 37]. Here, genistein up-regulated Bax and Bid levels, while down-regulating Bcl-2 and Bcl-xl expression, suggesting that the effects of genistein against esophageal cancer are multifaceted.

Mechanistically, in the current study, we found that the phosphorylation levels of Akt and JAK1/2 were significantly down-regulated in EsC cells following genistein treatment. EGFR, the common upstream activator of the Akt and JAK1/2 signaling pathways [38, 39] has tyrosine kinase catalytic activity, which can activate multiple downstream pathways, including the Akt and JAK1/2 signaling axis [40, 41]. Our results indicate that genistein, a tyrosine kinase inhibitor, not only reduced JAK1/2 and STAT3 (not STAT1) phosphorylation levels but also restricted STAT3 nuclear translocation. In addition, the down-regulation of Akt phosphorylation induced by genistein could reduce MDM2 phosphorylation levels [42]. The inactive MDM2 was unable to bind to P53 and mediate P53 degradation [43, 44], which may have contributed to the up-regulation in P53 levels. The phosphorylation of the core histone protein H2AX (γH2AX), a sensitive molecular marker of DNA double strand breaks [45], was greatly elevated with the increasing apoptotic ratio of EsC cells treated with genistein. Furthermore, the ATM-CHK2 and ATR-CHK1 pathways are activated by DNA damage [46], where the DNA damage response kinases ATM and ATR [47], and the serine/threonine kinase CHK2 [48] were phosphorylated and remarkably up-regulated in genistein-treated EsC cells. Furthermore, genistein induction following Akt or JAK1/2 pathway inhibitor treatment significantly attenuated gene expression associated with the cell cycle and facilitated apoptotic gene expression *in vitro* and *in vivo*.

Our results suggest that long-term treatment with genistein strikingly reduced EGFR expression, and genistein contributed to the reduced reactivity of EsC cells to EGF, which amplified the anti-tumor effect of genistein in EsC cells. Specifically, genistein inhibited EGFR-regulated downstream signaling molecules, leading to the restriction of cell proliferation. Previous studies have shown that EGFR is highly expressed in esophageal cancer and promotes its progression [49, 50]. This positive feedback effect may be more significant in esophageal cancer with high EGFR expression. However, whether genistein inhibits EGFR expression by modulating the Akt and JAK1/2 signaling pathways needs further investigation. Work in prostate cancer cells has shown that genistein treatment significantly up-regulated 33 miRNAs whereas miR-574-3p down-regulated EGFR expression by directly binding to the 3'UTR of EGFR, thereby inhibiting the malignant biological behavior of the tumor cells [51]. This provides new insights into the effects of genistein in esophageal cancer.

In summary, our findings revealed that genistein, a tyrosine kinase inhibitor, inhibits EGFR expression, resulting in the restriction of the Akt and JAK1/2 signaling pathways, thereby suppressing cell proliferation and inducing tumor cell apoptosis *in vitro* and *in vivo*. Genistein may be a promising adjuvant drug in the management of EsC.

## MATERIALS AND METHODS

### Cell lines and cell culture

Human esophageal cancer cell lines (Eca-109, EC9706, CaES-17) and the Het-1A human esophageal epithelial cell line were purchased from the Cell Bank of Shanghai Institutes for Biological Sciences, CAS. Cells were cultivated with RPMI1640 medium (Solarbio Technology Co., Ltd., Beijing, China) supplemented with fetal bovine serum (FBS) (10%) (Solarbio Technology Co., Ltd., Beijing, China) and penicillin-streptomycin (1%) (Solarbio Technology Co., Ltd., Beijing, China) in a humidified atmosphere at 37°C with 5% CO_2_. The culture medium was changed every 24 h.

### Cell proliferation assay

Cells were seeded into 96-well plates at a density of 5×10^3^ cells per well. Different concentrations of genistein (Sigma-Aldrich) were added 12 h later. Cell proliferation was measured using a CCK-8 reagent (Solarbio Technology Co., Ltd., Beijing, China) every 24 h, following the manufacturer’s protocol, and the CCK-8 reagent was added (to a final concentration of 10%) and then incubated at 37°C for 2 h. The absorbance at 450 nm was measured using a microplate reader (iMark, Bio-Rad).

### Cell clone formation

Cells were seeded into 6-well plates (Corning, Inc., NY, USA) at a density of 1×10^3^ cells per well, and subjected to different concentrations of genistein for 10 d. After washing with PBS, the cell clusters were fixed using 75% ethanol for 10 min and stained using 0.1% crystal violet for 20 min at room temperature. Each experiment was repeated three times. The number of cell clones in each well (n = 6) was counted directly using an optical microscope (Leica, Germany). After randomly selecting five fields of vision in each well, IPP 6.0 software was used to count the number of cells in each field of view (n = 10).

### Western blot analysis

The cells were lysed using RIPA lysis buffer (Beyotime Biotechnology, Shanghai, China) supplemented with phenylmethanesulfonyl fluoride (PMSF) (Beyotime Biotechnology, Shanghai, China). Proteins were collected from the supernatant after centrifugation at 12,000 g and 4°C for 10 min. The protein concentration was quantified using the BCA method. Proteins were fractionated through 10% sodium dodecyl-sulfate polyacrylamide gel electrophoresis (SDS-PAGE) (20 μg/per lane) and transferred onto polyvinylidene fluoride membranes (Millipore). Subsequently, the membranes were blocked using 5% milk in tris buffered saline containing 0.1% (v/v) Tween 20 (TBST) for 2 h at room temperature and incubated with corresponding primary antibodies (Table 1) for 2 h at room temperature. Afterwards, the membranes were incubated with horse anti-mouse (dilution 1:5000, #7076, Cell Signaling Technology) or goat anti-rabbit (dilution 1:5000, #7074, Cell Signaling Technology) horseradish peroxidase-linked secondary antibodies for 2 h at room temperature. The blots were visualized using an electrogenerated chemiluminescence detection kit (Millipore) and quantified using Quantity One V4.6.7 software.

**Table 1:** The corresponding primary antibodies used in this study.

**Genes**	**Manufacturer**	**Catalog number**	**Dilutions**
Bcl-2	Abcam	ab196495	1:3500
Bcl-xl	Abcam	ab199099	1:4000
Bax	Abcam	ab53154	1:6000
Bid	Abcam	ab32060	1:6000
PI3K	Abcam	ab32089	1:3500
p-Akt	Abcam	ab131443	1:1500
β-actin	Abcam	ab8227	1:5000
CyclinD1	Abcam	ab226977	1:4500
CDK4	Abcam	ab137675	1:4500
CDK6	Abcam	ab151247	1:4000
P53	Abcam	ab131442	1:4000
Ki-67	Abcam	ab15580	1:500
γH2AX	Abcam	ab26350	1:4500
ATM	Cell Signaling Technology (CST)	2873	1:3500
p-ATM	CST	13050	1:1500
ATR	CST	13934	1:3500
p-ATR	CST	2853	1:1500
CHK2	CST	2662	1:3500
p-CHK2	CST	2197	1:1500
JAK1	CST	3332	1:3500
p-JAK1	CST	74129	1:1500
JAK2	CST	74987	1:3500
p-JAK2	CST	3771	1:1200
STAT1	CST	9172	1:3500
p-STAT1	CST	7649	1:1300
STAT3	CST	12640	1:3500
p-STAT3	CST	4113	1:1600
MDM2	CST	86934	1:3500
p-MDM2	CST	3521	1:1800
Akt	CST	9272	1:3500
EGFR	CST	4267	1:1800
PARP	CST	9532	1:2000
p-PARP	CST	9548	1:2000
Caspase-3	CST	9662	1:2500
C-Caspase-3	CST	9664	1:2000

### qPCR analysis

Total RNA was extracted from the cells using TRIzol^®^ reagent (Thermo Fisher Scientific) following the manufacturer’s instructions. An A260/A280 ratio in the range of 1.8–2.0 was considered to indicate acceptable purity. Subsequently, 1 μg of total RNA was reverse transcribed into cDNA at 37°C for 15 min, 85°C for 5 sec, and then stored at 4°C. qPCR was performed using the ABI7500 Real-Time PCR System (Applied Biosystems) and a SYBR Green PCR kit (Takara, Otsu, Japan). GAPDH was chosen as the internal reference. The sequences of all primers are listed in Table 2. The thermocycling procedures are shown as follows: pre-denaturation at 95°C for 2 min; denaturation at 95°C for 15 sec; annealing at 60°C for 15 sec; all for 40 cycles. Each sample was measured in triplicate. Gene expression was calculated using the 2^-ΔΔCt^ method.

**Table 2:** Real time PCR primers used in this study.

**Genes**	***Forward***	***Reverse***
*Bcl-2*	5′-CCAGCGTATATCGGAATGTGG-3′	5′-CCATGTGATACCTGCTGAGAAG-3′
*Bcl-xl*	5′-GAGCTGGTGGTTGACTTTCTC-3′	5′-TCCATCTCCGATTCAGTCCCT-3′
*Bax*	5′-CCCGAGAGGTCTTTTTCCGAG-3′	5′-CCAGCCCATGATGGTTCTGAT-3′
*Bid*	5′-ATGGACCGTAGCATCCCTCC-3′	5′-GTAGGTGCGTAGGTTCTGGT-3′
*PARP*	5′-GTGGTCGGGACTGTCTCTAAG-3′	5′-TCTCCAGTAGCAACCTGAAAAGT-3′
*Caspase-3*	5′-AGAGGGGATCGTTGTAGAAGTC-3′	5′-ACAGTCCAGTTCTGTACCACG-3′
*CyclinD1*	5′-TGGAGCCCGTGAAAAAGAGC-3′	5′-TCTCCTTCATCTTAGAGGCCAC-3′
*CDK4*	5′-TTCGTGAGGTGGCTTTACTG-3′	5′-GATATGTCCTTAGGTCCTGGTCT-3′
*CDK6*	5′-TCTTCATTCACACCGAGTAGTGC-3′	5′-TGAGGTTAGAGCCATCTGGAAA-3′
*P53*	5′-GTTTCCGTCTGGGCTTCTTG-3′	5′-CACAACCTCCGTCATGTGCT-3′
*ATM*	5′-TTGATCTTGTGCCTTGGCTAC-3′	5′-TATGGTGTACGTTCCCCATGT-3′
*ATR*	5′-AGCAGCGATACTGTTGAATGTG-3′	5′-TGCCTCGATGAGGAAAACCAC-3′
*CHK2*	5′-TGAGAACCTTATGTGGAACCCC-3′	5′-ACAGCACGGTTATACCCAGC-3′
*JAK1*	5′-CTTTGCCCTGTATGACGAGAA-3′	5′-ACCTCATCCGGTAGTGGAG-3′
*JAK2*	5′-ATCCACCCAACCATGTCTTCC-3′	5′-ATTCCATGCCGATAGGCTCTG-3′
*STAT1*	5′-CGGCTGAATTTCGGCACCT-3′	5′-CAGTAACGATGAGAGGACCCT-3′
*STAT3*	5′-ACCAGCAGTATAGCCGCTTC-3′	5′-GCCACAATCCGGGCAATCT-3′
*MDM2*	5′-TCCTGTAGTTTCGTCAGATCCT-3′	5′-CCGTTTCAATCGGGATACTTCA-3′
*PI3K*	5′-AGAGCACTTGGTAATCGGAGG-3′	5′-CTTCCCCGGCAGTATGCTTC-3′
*Akt*	5′-AGCGACGTGGCTATTGTGAAG-3′	5′-GCCATCATTCTTGAGGAGGAAGT-3′
*EGFR*	5′-TTGCCGCAAAGTGTGTAACG-3′	5′-GTCACCCCTAAATGCCACCG-3′
*β-actin*	5′-CCTCGCCTTTGCCGATCC-3′	5′-GGATCTTCATGAGGTAGTCAGTC-3′

### Xenograft assays

The animal experiments in the current study were approved by the Institutional Animal Care and Use Committee of the First Affiliated Hospital of Xiamen University (approval number: XMU-AEA-20180137). Cells at the logarithmic growth phase were digested, resuspended in PBS to 2×10^7^ cells/mL, and mixed with an equal volume of Matrigel (BD Biosciences). The cell suspension (100 μL; containing 1×10^6^ cells) was inoculated subcutaneously in the back of the hind limbs of nude mice. For genistein treatment, nude mice were randomly divided into three groups (n = 6 for each group). Then, 1 week later, 5 mg/kg or 10 mg/kg of genistein or an equal volume of PBS was administered through gavage every 2 d. Additionally, for genistein combined with the JAK1 pathway inhibitor GLPG0634 or the Akt pathway inhibitor MK-2206, nude mice were randomly divided into three groups (n = 6 for each group). Then, 1 week later, 5 mg/kg of genistein plus 1 mg/kg of GLPG0634 or 10 mg/kg of genistein plus 1 mg/kg of MK-2206 or an equal volume of PBS were administered through gavage every 2 d. The tumor diameter was measured weekly. Animals were sacrificed through cervical dislocation in a manner consistent with animal ethics after 42 d, and tumor tissues were carefully excised to detect tumor volume (V). The tumor volume was estimated using the formula: 0.5 × length × (width)^2^.

### ROS measurement

An oxidative stress indicator CM-H2DCFDA (Solarbio Technology Co., Ltd., Beijing, China) was added after the cells were treated with genistein, followed by incubation at 37°C for 30 min in a cell culture incubator. Next, the cells were washed with PBS twice and digested to create a cell suspension. A total of 100 μL of the cell suspension was centrifuged at 200×g for 5 min; the supernatant was discarded and the cells were resuspended in PBS containing 2% FBS. The cells were washed with PBS twice and then resuspended in 200 μL of PBS containing 2% FBS. The fluorescence intensities of the cells were detected through flow cytometry (Guava EasyCyte TM 8HT, Merk).

### Cell cycle and apoptosis assays

Cells at the logarithmic growth phase were seeded into 6-well plates (5×10^5^ cells/well). After treatment with genistein for 24 h, the cells were collected and washed twice with pre-cooled PBS, and then centrifuged at 200×g for 10 min. After the PBS was discarded, the cells were fixed in pre-cooled 70% ethanol at 4°C overnight. Next, propidium iodide (PI) (Solarbio Technology Co., Ltd., Beijing, China) staining was performed for 30 min at room temperature in the dark. Subsequently, the cell cycle was assessed through flow cytometry (Guava EasyCyte TM 8HT, Merk). For cell apoptosis, the cells were washed twice with pre-cooled PBS, and then incubated with 10 μL of Annexin V-FITC (Solarbio Technology Co., Ltd., Beijing, China) and 5 μL of PI (0.25 mg/mL) for 20 min at room temperature in the dark. Flow cytometry was used to detect apoptotic cells.

### Mitochondrial membrane potential detection

Cells at the logarithmic growth phase were seeded into 6-well plates (5×10^5^ cells/well). After treatment with different concentrations of genistein, 10 μg/mL of JC-1 solution (Solarbio Technology Co., Ltd., Beijing, China) was added. The cells were incubated at 37°C for 15 min, washed with PBS three times, digested in 0.25% trypsin (Gibco, Thermo Fisher Scientific) and centrifuged at 200×g for 10 min. The mitochondrial membrane potential was measured through flow cytometry. When the mitochondrial membrane potential is low, JC-1 is green. In contrast, JC-1 shifts to red when the mitochondrial membrane potential is high.

### Cytoplasmic and nuclear protein extraction

NE-PER^TM^ nuclear and cytoplasmic extraction reagents (cat. no. 78833, Invitrogen) were used for cytoplasmic and nuclear protein extraction. Cells at the logarithmic growth phase were seeded into 6-well plates (5×10^5^ cells/well) and treated with different concentrations of genistein for 72 h. Afterwards, the cells were collected and vortexed vigorously for 15 seconds. The suspended cells were incubated with pre-cooled Cytoplasmic Extraction Reagent (CER) I on ice for 10 min. Next, pre-cooled CER II reagent was added, and the samples were vortexed for 5 sec, placed on ice for 1 min, vortexed for 5 sec again, and centrifuged at 14,000×g for 5 min (4°C). The supernatant (cytoplasmic fraction) was immediately transferred into a clean pre-chilled EP tube and then stored at −80°C for western blotting. In addition, the insoluble precipitate (nuclei) was resuspended in pre-cooled Nuclear Extraction Reagent for 40 min on ice, with vortexing at 10 min intervals, followed by centrifugation at 14,000×g for 10 min (4°C). The supernatant (nuclear protein) was prepared for western blotting.

### Statistical analysis

Data analysis and graphical representation were performed using SPSS 21.0 (IBM Corporation, USA) and GraphPad Prism 6.0 (GraphPad Software, USA), respectively. The difference between two groups was tested using the Student’s t-test, and comparisons among multiple groups were measured using one-way ANOVA with Dunnett’s test or Fisher’s least significant difference (LSD) test. Each experiment was independently repeated in triplicate, and *P*<0.05 was considered to indicate statistical significance.

## Supplementary Material

Supplementary Figures
