# Supplementary material for: Inhibition of esophageal-carcinoma cell proliferation by genistein via suppression of JAK1/2-STAT3 and AKT/MDM2/p53 signaling pathways
Source: Aging (Albany NY). 2020 Apr 10;12(7):6240–59. doi: 10.18632/aging.103019 (PMC7185096; doi:10.18632/aging.103019)
Supplement: Supplementary Figures [file aging-12-103019-s001..pdf]

SUPPLEMENTARY FIGURES

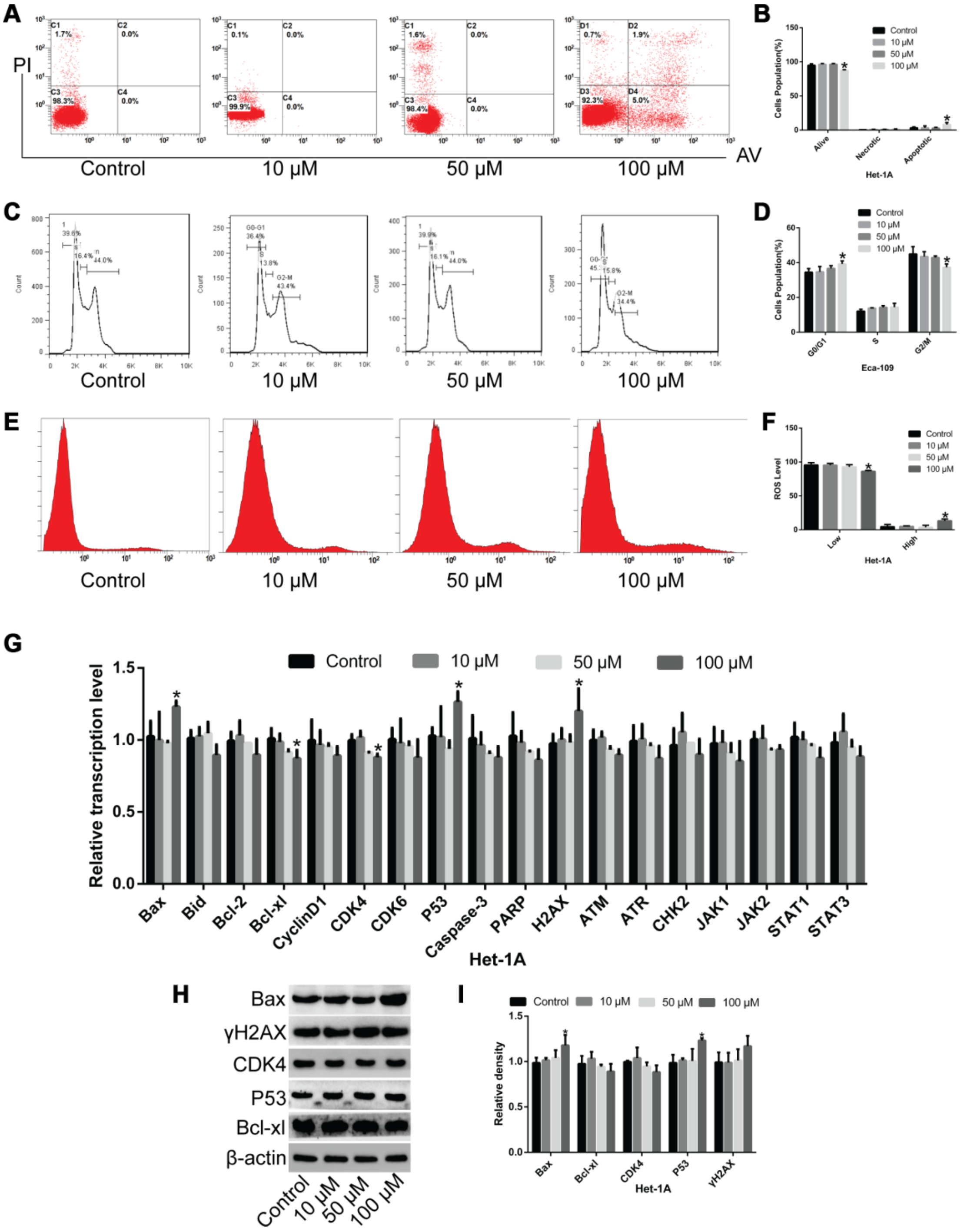

Supplementary Figure 1: The effect of genistein treatment on human esophageal epithelial Het-1A cells. (**A**, **B**) The apoptotic rate, (**C**, **D**) the cell cycle distribution and (**E**, **F**) ROS level of Het-1A cells treated with various concentrations of genistein for 24 h. (**G**) The mRNA levels of Bax, Bid, Bcl-xl, CyclinD1, CDK4, CDK6, P53, Caspase-3, PARP, H2AX, ATM, ATR, CHK2, JAK1, JAK2, STAT1 and STAT3 in Het-1A cells treated with various concentrations of genistein for 72 h. (**H**, **I**) Protein levels of Bax, H2AX, CDK4, P53 and Bcl-xl in Het-1A cells treated with various concentrations of genistein for 72 h. All in vitro experiments were independently repeated three times. Comparisons among multiple groups were measured using one-way ANOVA with Dunnett's test. Data are presented as the mean ± SD. **P*<0.05.

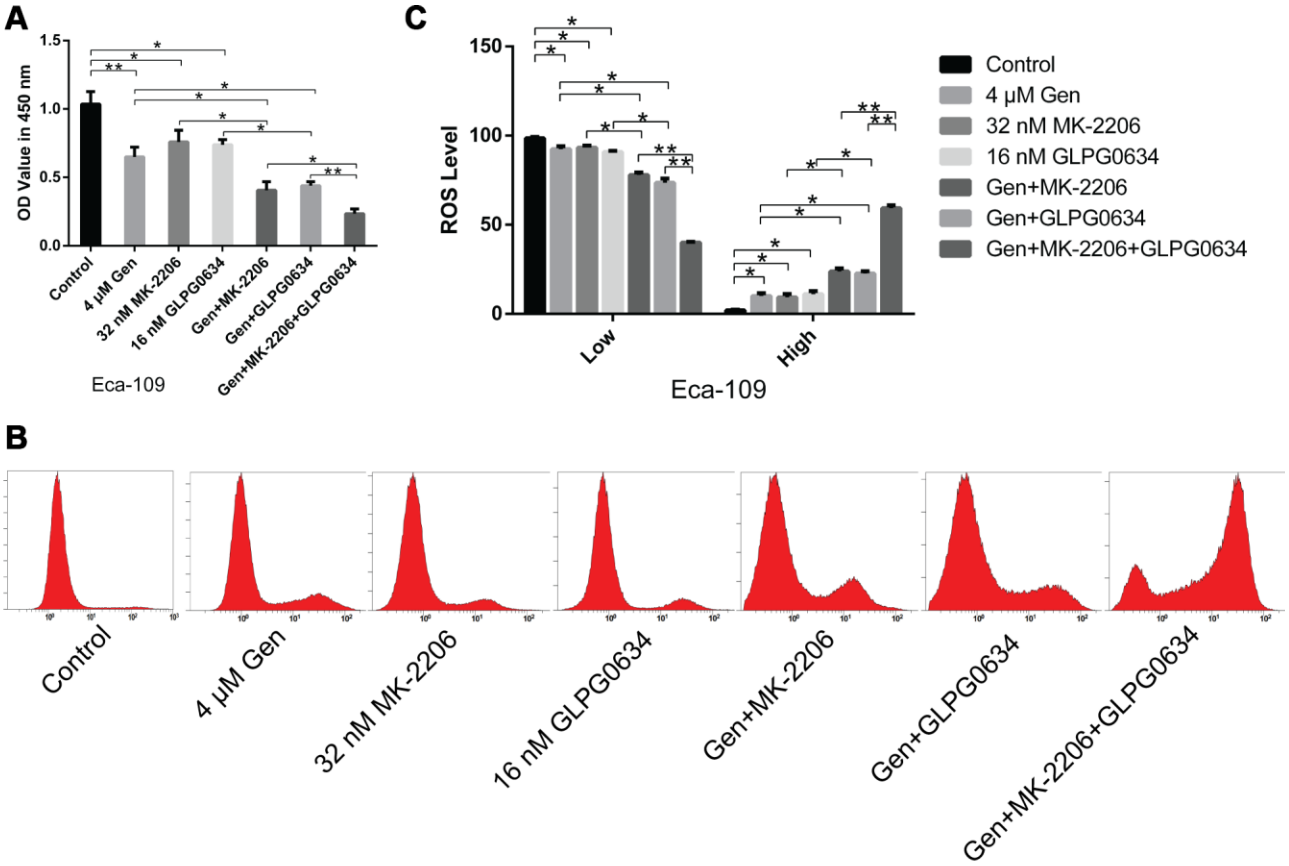

Supplementary Figure 2. The synergistic effect of genistein with JAK1 and Akt pathway inhibitors on Eca-109 cells. Genistein (4 μM) plus JAK1 pathway inhibitor GLPG0634 (16nM) and Akt pathway inhibitor MK-2206 (32nM) could extremely decreased (**A**) cell viability and facilitated (**B**, **C**) ROS production in comparison to Eca-109 cells treated with genistein and in combination with GLPG0634 or MK-2206 for 72 h. All in vitro experiments were independently repeated three times. Comparisons among multiple groups were measured using one-way ANOVA with Dunnett's test. Data are presented as the mean ± SD. *P<0.05; **P<0.01; Gen, genistein.
